# Mucocutaneous Manifestations of HIV and the Correlation with WHO Clinical Staging in a Tertiary Hospital in Nigeria

**DOI:** 10.1155/2014/360970

**Published:** 2014-12-21

**Authors:** Olumayowa Abimbola Oninla

**Affiliations:** Department of Dermatology and Venereology, Faculty of Clinical Sciences, College of Health Sciences, Obafemi Awolowo University, P.O. Box 2545, Ile-Ife 250005, Osun State, Nigeria

## Abstract

Skin diseases are indicators of HIV/AIDS which correlates with WHO clinical stages. In resource limited environment where CD4 count is not readily available, they can be used in assessing HIV patients. The study aims to determine the mucocutaneous manifestations in HIV positive patients and their correlation with WHO clinical stages. A prospective cross-sectional study of mucocutaneous conditions was done among 215 newly diagnosed HIV patients from June 2008 to May 2012 at adult ART clinic, Wesley Guild Hospital Unit, OAU Teaching Hospitals Complex, Ilesha, Osun State, Nigeria. There were 156 dermatoses with oral/oesophageal/vaginal candidiasis (41.1%), PPE (24.4%), dermatophytic infections (8.9%), and herpes zoster (3.8%) as the most common dermatoses. The proportions of dermatoses were 4.5%, 21.8%, 53.2%, and 20.5% in stages 1–4, respectively. A significant relationship (using Pearson's Chi square with *P* value <0.05) was obtained between dermatoses and WHO clinical stages. Pearson's correlation coefficient showed a positive correlation between the number of dermatoses and the WHO clinical stages. Dermatoses can therefore serve as diagnostic and prognostic markers in resource limited settings to initiate HAART in clinical stages 3 and 4.

## 1. Introduction

The burden of skin disease in developing countries particularly the sub-Saharan Africa is high with a serious impact on the quality of life and resulting loss of productivity at work and school and disfigurement [1–4]. Infectious dermatoses particularly superficial fungal infections, scabies, and impetigo are the most common skin problems due to overcrowding with a hot and humid environment, poor sanitary conditions, sharing of personal effects or fomites, and poor access to medical supplies and treatment [5, 6].

The skin problems here are further compounded by the high prevalence of HIV which commonly causes skin lesions [7]. It was reported that approximately 90% of people living with HIV have skin changes and symptoms during the course of their disease [8]. Skin diseases are significantly higher among HIV positive than HIV negative individuals [9]. Differences in skin pigmentation, climate, hygiene, and genetic, environmental, demographic, and behavioral factors cause different clinical presentations and epidemiologic patterns of HIV-associated skin disease in Africa [10, 11].

Skin findings are regarded by WHO as useful in assessing severity of HIV infection in patients in resource limited environment [12].

Knowledge of the skin and mucosal signs of HIV/AIDS is important, as mucocutaneous lesions are usually the first manifestation of HIV, ensures early diagnosis and prompt treatment, and reveals complications as HIV causes atypical and severe presentations of these conditions [13, 14]. Those involved in health care of HIV patients must therefore know the type, pattern, and prevalence of skin diseases in their locality [15–17]. Mucocutaneous diseases have been correlated with CD4 counts in many studies [18], while few studies (and none in this country to the knowledge of this researcher) documented the clinical correlation of these diseases to WHO clinical stages [19–21].

Although HIV dermatoses have been widely documented, reports of the type of dermatoses in HIV patients in this country are few and nonexistent in this area of study [21, 22]. This center was one of the recently established adult HIV care center with facility for diagnosis and care for HIV patients and in collaboration with the Institute of Human Virology center in OAUTHC, Ile-Ife. The aim of this study is to determine the cutaneous skin markers in HIV in an adult HIV clinic in Nigeria. The objective is to identify and correlate these mucocutaneous disorders to the clinical stages at time of presentation for HIV care.

## 2. Methodology

A preliminary study of skin diseases was conducted in a newly created adult ART clinic as a prospective cross-sectional study among 215 newly diagnosed adult HIV patients. Consecutive patients presenting at the adult ART clinic of Wesley Guild Hospital Unit of Obafemi Awolowo University Teaching Hospitals Complex (OAUTHC) in Ilesha from June 2008 to May 2012 were studied.

Patients, who reported for care after testing HIV-positive at our screening centre, were included in the study. The skin symptoms were documented, and detailed clinical examination of the skin in broad daylight was carried out. Skin findings were recorded, and patients were categorized according to the WHO clinical stages at presentation using skin and findings in other body systems.

Excluded were old patients of the unit prior to onset of the study who are still receiving HIV care; those who came from other HIV centres to continue their HIV care at this centre due to proximity or other reasons; referred patients who had started HAART; patients on any primary or secondary prophylaxis for opportunistic infections; and patients on any other medication for systemic diseases. This is to prevent possible alteration in skin pathologies reported.

Diagnoses were mainly clinical and when necessary mycological, histopathological, and hematological tests were carried out. Ethical approval for the study was obtained from OAUTHC Research and Ethics Committee.

## 3. Results

There were 71 males and 144 females with a ratio of 1 : 2. The age ranges between 18 and 77 with a mean of 35 years. A total of 215 new cases of HIV positive patients were seen, and 113 had symptoms and signs of mucocutaneous disease on their first day of presentation at the adult HIV clinic. Patients with one, two, or three skin diseases were 76, 31, and 6, respectively.

The total skin conditions found were 156 with oral and oesophageal candidiasis, pruritic papular eruption, vaginal candidiasis, dermatophytic infections, herpes zoster, viral warts, herpes simplex, carbunculosis/folliculitis barbae, and seborrhoeic dermatitis as the most common dermatoses. Eosinophilic folliculitis and psoriasis were uncommon. No case of Kaposi sarcoma was recorded. Infectious dermatoses constituted 65.2% (fungal 50%, viral 12%, and bacterial 3.2%) of all dermatoses and noninfectious skin conditions 34.8% (see Table 1).

There were 68 patients in WHO clinical stage 1, 31 in stage 2, 87 in stage 3, and 29 in stage 4. The percentage of patients in each stage who had skin diseases was 8.8%, 77.4%, 71.3%, and 72.4%, respectively. Pruritic papular eruptions were the most common condition in stages 1 and 2, while oral/oesophageal thrush was more in stages 3 and 4. The different skin diseases and the clinical stages at diagnosis of the disease are shown in Table 2. The presence of skin disease was significantly associated with the clinical stage of the patient (see Table 2).

The proportion of the total number (156) of cutaneous diseases occurring in stage 1 was 4.5% and 21.8% in stage 2, 53.2% in stage 3, and 20.5% in stage 4 (see Table 2). There was a significant relationship using Pearson's Chi square with *P* value <0.05 and Pearson's correlation coefficient between the number of skin diseases that a patient has and the clinical stage at presentation (see Table 3).

## 4. Discussion

Skin diseases act as indicators of HIV and AIDS. The clinical diagnoses of skin diseases have been found to correlate with histopathological findings even in HIV patients [23]. Hence, good clinical acumen is essential to make correct diagnoses of skin problems in these patients.

The majority of the patients were females. A higher female preponderance was also reported by Salami et al. in Nigeria and Glynn et al. in Kenya [24, 25]. The greater susceptibility of females to HIV may explain the gender differences in HIV prevalence [25]. More than half (52.6%) of the newly diagnosed HIV patients had dermatoses. A similar study by Mawenzi et al. [26] among newly diagnosed HIV patients in Kenya reported a prevalence of 42.1%. Nnoruka et al. [27] obtained a prevalent rate of 93.5% in southeast Nigeria. In Sanandaj city, Pakistan, it was as high as 94.3% [28].

Infectious dermatoses made up 65.2% of the dermatoses (50% fungi, 12% viral, and 3.2% bacterial) and are comparable to 64.3% reported by Salami et al. in which viral skin conditions accounted for 37.1% and fungal infections, 24.3% [24]. Another Nigerian study by Nnoruka [21] found skin infections as the most common dermatoses, while they were the second in occurrence in a report by Yahya in northern Nigeria [22]. In some developed countries, infections still rank prominent as one of the most prevalent dermatoses in HIV [29]. Most infectious dermatoses start in early stages (stage 1) increasing in number, becoming more diffuse and more resistant to treatment as disease progresses [18, 27]. In this study, infectious diseases were found mostly in stages 3 and 4. A similar report was obtained in Nigerian children by Okechukwu et al. [30]. This implies that skin infections are commonly associated with HIV infection.

The most common skin disorders in this study were oropharyngeal candidiasis (32.1%), pruritic papular eruptions (25.3%), vaginal candidiasis (9.0%), dermatophytic infections (8.9%), herpes zoster (3.9%), carbunculosis (2.6%), herpes simplex (2.6%), and seborrhoeic dermatitis (1.9%). Papulopruritic eruption, seborrheic dermatitis (35.8%), herpes zoster, dermatophytosis (24.3%), oropharyngeal candidiasis, staphylococcal infection, and vaginal candidiasis were the most common in southeast Nigeria [27].

Sivayathorn et al. in Bangkok found that oral candidiasis (34.3%), pruritic papular eruption (PPE) (32.7%), seborrhoeic dermatitis (21%), herpes zoster (16.1%), oral hairy leukoplakia (14.9%), herpes simplex (10.9%), onychomycosis (9.3%), cutaneous ringworm (7.7%), psoriasis (6.5%), and folliculitis (5.6%) were the most prevalent skin conditions among HIV seropositives [31].

Thompson et al. in Jamaica, a developing and tropical country like Nigeria, reported that papular prurigo, oral candidiasis, dermatophyte infections, herpes simplex infections, and seborrhoeic dermatitis were the most frequently diagnosed dermatological disorders, while Kaposi sarcoma is relatively rare [32]. These studies showed that skin diseases that commonly affect HIV patients in Nigeria are similar to other parts of Africa and the world. They therefore are related to the immunopathological changes due to HIV infection.

Mucocutaneous findings in this cohort of HIV positive patients correlate well with the WHO clinical stages. Few patients in stage 1 had skin lesions and their skin lesions were nonspecific. A significant increase in number of dermatoses was observed as the clinical stages progressed. This is similar to an earlier report by Wiwanitkit [33]. This is also buttressed by a Nigerian study by Nnoruka et al. [27] which showed a positive correlation between mucocutaneous manifestations of HIV and low CD4 counts. Sivayathorn et al. [31] found a significantly more number of skin disorders in patients in stages 2 and 3 subgroups than stage 1.

Pruritic papular eruption (PPE) was the most common dermatosis in stages 1 and 2 and next to oral candidiasis in stages 3 and 4. The majority of the affected patients, however, were in stages 3 and 4. Nnoruka et al. [27] found it to occur in those with lower immune status with CD4 count that was less than 200 cells/mm^3^. Mawenzi et al. and Goldstein et al. observed a significant relationship between pruritic papular eruption and low CD4 counts [26, 34]. Boonchai et al. [35] studied PPE in HIV and reported it as a cutaneous skin marker of advanced HIV infection. PPE can be used as a clinical indicator for HAART in resource limited environments [26].

Oral thrush was the predominant dermatoses in stage 3 and stage 4. Oral thrush particularly if extending to the esophagus has been widely reported to be synonymous with severe immunosuppression [36]. In Nigeria, the prevalence of oral candidiasis ranges between 36 and 80% in HIV patients [37, 38], and the condition is predictive of severe immunosuppression with approximately 50% of patients developing AIDS within 5 years [39]. Goh et al. [11], Sharma et al. [40], and Puttaiah et al. [41] reported lower CD4 counts less than 200 cells/mm^3^ in patients with oral thrush. Vaginal candidiasis also occurred in stage 3. It was found to occur concomitantly with oral candidiasis and AIDS defining medical conditions [26]. It can therefore be a clinical indicator of advanced HIV infection.

Dermatophytic infections were found mostly in stages 3 and 4. Extensive dermatophytosis was found in 26% of HIV patients seen by Nnoruka in southeast Nigeria though the clinical stages were not stated [21]. They are often among the top ten dermatoses in HIV [24, 33].

Herpes zoster was seen mostly in stage 2. The WHO classified it as a stage 2 disease [19]. Herpes zoster is associated with early immunosuppression [27], and a significant change occurs in the clinical stage with disease progression [16].

Viral warts occurred more in stages 2 and 3. About 13% of HIV patients seen at Irua [24] in Nigeria had plane and anogenital warts though the relationship to clinical stages was not known. They were found to occur at CD4 counts of 200–300 cells/mm^3^. Mawenzi et al. [26] report showed that they were associated with higher immune status in HIV (CD4 >300 cells/mm^3^). They have been listed as a stage 2 clinical disease by WHO.

Bacterial skin infections occurred in those in stages 2 and 3. They have been reported to be common in HIV patients but correlation with clinical stages is not known though Nnoruka et al. report them to occur in those with CD4 counts between 200 and 500 cells/mm^3^ [27]. Study by Smith et al. [20] showed a peak occurrence in early and midstage disease with a decreased occurrence in the late stage.

Seborrhoeic dermatitis has been listed as a stage 2 disease by WHO. In this study, the prevalence was low and it was found in those in stages 2 and 3. Nnoruka et al. [27] found a positive correlation between seborrhoeic dermatitis and CD4 counts of 200–500 cells/mm^3^. Salami et al. [24], however, showed the occurrence to be in patients with lower immune status (CD4 counts <100 cells/mm^3^). The differences were adduced to be as a result of variable level of severity of seborrhoeic dermatitis [26].

## 5. Conclusion

HIV-related mucocutaneous manifestations are very common and with good clinical acumen are easily diagnosed. Oropharyngeal candidiasis, pruritic papular eruption, vaginal candidiasis, dermatophytic infections, herpes zoster, carbunculosis, herpes simplex, and seborrhoeic dermatitis significantly correlated with WHO clinical stages.

Dermatoses can therefore serve as diagnostic and prognostic markers in a resource limited setting as obtained in many HIV clinics in Nigeria to stage the severity of HIV infection and to initiate HAART for those in clinical stages 3 and 4 where laboratory tests for CD4 count are not available. Planning for HIV care in this environment should also inculcate plans for skin diseases which affect them more than the general populace.

## Figures and Tables

**Table 1:** Skin diseases in HIV patients according to gender.

Skin diseases in HIV patients	Gender		Total
	Male	Female	
*Infectious dermatoses *			65.2%
Viral:			
anogenital warts/verruca plana	2	4	6
herpes simplex labialis/genitalis	1	4	5
herpes zoster	3	3	6
molluscum contagiosum	1	0	1
mumps	0	1	1
Bacterial:			
carbunculosis/folliculitis barbae	2	3	5
Fungi:			
oral thrush/oesophageal thrush	14	36	50
tinea faciae	1	0	1
tinea corporis	1	6	7
tinea manuum	0	1	1
tinea unguium	2	3	5
vaginal candidiasis	0	14	14
Parasitic:			
Loa loa and elephantiasis	2	0	2
*Noninfectious dermatoses *			34.8%
angular stomatitis	1	0	1
eosinophilic folliculitis	2	0	2
miliaria rubra	0	1	1
pruritic papular eruptions	15	23	38
pruritus	1	1	2
psoriasis	0	2	2
seborrhoeic dermatitis	1	2	3
scar	0	1	1
striae	0	1	1
xerosis	0	1	1
No skin disease	32	70	102

Total	71 (33%)	144 (67%)	215 (100.0%)

**Table 2:** Dermatoses occurring in each WHO clinical stage.

Dermatoses	WHO clinical staging at diagnosis of skin disease				Total	Percentage (%)
	1	2	3	4		
Carbunculosis/folliculitis barbae	1	2	2	0	5	3.2
Eosinophilic folliculitis	0	0	1	1	2	1.3
Herpes simplex	0	2	2	1	5	3.2
Herpes zoster	0	4	2	0	6	3.8
Loa loa and elephantiasis	0	1	1	0	2	1.3
Molluscum contagiosum	0	0	1	0	1	0.6
Miliaria rubra	0	1	0	0	1	0.6
Mumps	0	0	0	1	1	0.6
Pruritic papular eruptions	2	11	16	9	38	24.4
Pruritus	0	2	0	0	2	1.3
Psoriasis	0	1	1	0	2	1.3
Scar	1	0	0	0	1	0.6
Seborrhoeic dermatitis	0	1	2	0	3	1.9
Angular stomatitis	0	0	0	1	1	0.6
Striae	1	0	0	0	1	0.6
Oral/oesophageal thrush	0	4	34	12	50	32.1
Tinea corporis	0	0	4	3	7	4.5
Tinea faciae	0	0	0	1	1	0.6
Tinea manuum	0	0	1	0	1	0.6
Tinea unguium	0	1	2	2	5	3.2
Vaginal candidiasis	1	2	11	0	14	9.0
Verruca plana/anogenital warts	1	2	2	1	6	3.8
Xerosis	0	0	1	0	1	0.6
Number of all skin lesions	7	34	83	32	156	100%
	(4.5%)	(21.8%)	(53.2%)	(20.5%)	(100%)	
Presence of skin disease						
Yes	6	24	62	21	113	52.6
No	62	7	25	8	102	47.4
	68	31	87	29	215	100%
	(31.6%)	(14.4%)	(40.5%)	(14.5%)	(100%)	

*χ* ^2^ = 76.641; df = 3; *P* = 0.000.

**Table 3:** Number of dermatoses in relation to WHO clinical stages.

Number of dermatoses	WHO staging at the time of diagnosis of skin disease				Total (%)
	1	2	3	4	
0	62	7	25	8	102 (47.4)
1	5	15	45	11	76 (35.3)
2	1	7	14	9	31 (14.4)
3	0	2	3	1	6 (2.8)

Total	68	31	87	29	215 (100)

*χ* ^2^ = 82.131; df = 9; *P* = 0.000.

^**^Pearson's correlation coefficient = 0.449; *P* = 0.000.

^**^Correlation is significant at the 0.01 level (2-tailed).

## References

[1] Kingman S. (2005). Growing awareness of skin disease starts flurry of initiatives. *Bulletin of the World Health Organization*.

[2] Satimia F. T., McBride S. R., Leppard B. (1998). Prevalence of skin disease in rural Tanzania and factors influencing the choice of health care, modern or traditional. *Archives of Dermatology*.

[3] Porter M. J. (1978). Problems and priorities for dermatology in developing countries. *International Journal of Dermatology*.

[4] Hay R. J., Steer A. C., Engelman D. (2012). Scabies in the developing world—its prevalence, complications, and management. *Clinical Microbiology and Infection*.

[5] Oninla O. A., Onayemi O. (2012). Skin infections and infestations in prison inmates. *International Journal of Dermatology*.

[6] Porter M. J. (1977). An epidemiological approach to skin disease in the tropics. *Tropical Doctor*.

[7] Hu J., McKoy K., Papier A., Klaus S., Ryan T., Grossman H., Masenga E. J., Sethi A., Craft N. (2011). Dermatology and HIV/AIDS in Africa. *Journal of Global Infectious Diseases*.

[8] Dlova N., Mosam A. (2004). Cutaneous manifestations of HIV/AIDS: part 1. *Southern African Journal of HIV Medicine*.

[9] Bakari M., Lyamuya E., Mugusi F., Aris E., Chale S., Magao P., Josiah R., Moshi A., Swai A., Pallangyo N., Sandstrom E., Mhalu F., Biberfeld G., Pallangyo K. (2003). The prevalence and pattern of skin diseases in relation to CD4 counts among HIV-infected police officers in Dar es Salaam. *Tropical Doctor*.

[10] Amerson E. H., Maurer T. A. (2010). Dermatologic manifestations of HIV in Africa. *Topics in HIV Medicine*.

[11] Goh B.-K., Chan R. K. W., Sen P., Theng C. T. S., Tan H.-H., Wu Y.-J., Paton N. I. (2007). Spectrum of skin disorders in human immunodeficiency virus-infected patients in Singapore and the relationship to CD4 lymphocyte counts. *International Journal of Dermatology*.

[12] WHO WHO case definitions of HIV for surveillance and revised clinical staging and immunological classification of HIV-related disease in adults and children. http://www.who.int/hiv/pub/vct/hivstaging/en/.

[13] Jordaan H. F. (2008). Common skin and mucosal disorders in HIV/AIDS. *South African Family Practice*.

[14] Kovarik C. L., Kekitiinwa A., Schwarzwald H. Cutaneous manifestations of HIV infection. http://www.bipai.org/HIV-curriculum/.

[15] Muñoz-Pérez M. A., Rodriguez-Pichardo A., Camacho F., Colmenero M. A. (1998). Dermatological findings correlated with CD4 lymphocyte counts in a prospective 3 year study of 1161 patients with human immunodeficiency virus disease predominantly acquired through intravenous drug abuse. *British Journal of Dermatology*.

[16] Smith K. J., Skelton H. G., Yeager J. (1994). Cutaneous findings in hiv-1-positive patients: a 42-month prospective study. military medical consortium for the advancement of retroviral research (MMCARR). *Journal of the American Academy of Dermatology*.

[17] Grant A. D., Djomand G., De Cock K. M. (1997). Natural history and spectrum of disease in adults with HIV/AIDS in Africa. *AIDS*.

[18] Raju P. V., Rao G. R., Ramini T. V., Vandana S. (2005). Skin disease: clinical indicator of immune status in human immunodeficiency virus (HIV) infection. *International Journal of Dermatology*.

[19] World Health Organization (2006). *WHO Case Definitions of HIV for Surveillance and Revised Clinical Staging and Immunological Classification of HIV-Related Disease in Adults and Children*.

[20] Smith K. J., Skelton H. G., Yeager J. (1994). Cutaneous findings in HIV-1-positive patients: a 42-month prospective study. Military Medical Consortium for the Advancement of Retroviral Research (MMCARR). *Journal of the American Academy of Dermatology*.

[21] Nnoruka E. N. (2005). Skin diseases in south-east Nigeria: A current perspective. *International Journal of Dermatology*.

[22] Yahya H. (2007). Change in pattern of skin disease in Kaduna, north-central Nigeria. *International Journal of Dermatology*.

[23] Halder S., Banerjee S., Halder A., Pal P. R. (2012). Skin diseases in HIV-infected patients: impact of immune status and histological correlation. *Indian Journal of Sexually Transmitted Diseases*.

[24] Salami T. A. T., Adewuyi G. M., Echekwube P., Affusim C. (2013). Pattern of cutaneous pathology among a cohort of HIV/AIDS patients accessing care in a rural/suburban adult ART clinic in Nigeria. *The British Journal of Medicine & Medical Research*.

[25] Glynn J. R., Caraël M., Auvert B., Kahindo M., Chege J., Musonda R., Kaona F., Buvé A. (2001). Why do young women have a much higher prevalence of HIV than young men? A study in Kisumu, Kenya and Ndola, Zambia. *AIDS*.

[26] Mawenzi R. L., Oguttu O. R., Williams H. C., Joash A. (2013). Epidemiology and clinical spectrum of cutaneous diseases manifesting among newly diagnosed HIV seropositive adults in Nakuru county-Kenya. *Continental Journal of Medical Research*.

[27] Nnoruka E. N., Chukwuka J. C., Anisuiba B. (2007). Correlation of mucocutaneous manifestations of HIV/AIDS infection with CD4 counts and disease progression. *International Journal of Dermatology*.

[28] Rad F., Ghaderi E., Moradi G., Mafakheri L. (2008). The relationship between skin manifestations and CD4 counts among HIV positive patients. *Pakistan Journal of Medical Sciences*.

[29] Garbe C., Husak R., Orfanos C. E. (1994). HIV-associated dermatoses and their prevalence in 456 HIV-infected patients. Relation to immune status and its importance as a diagnostic marker. *Der Hautarzt*.

[30] Okechukwu A. A., Okechukwu O. I., Jibril P. (2014). Patterns of skin disorder and its relationship with CD4^+^ cell count in a cohort of HIV-infected children. *Journal of Medicine and Medical Sciences*.

[31] Sivayathorn A., Srihra B., Leesanguankul W. (1995). Prevalence of skin disease in patients infected with human immunodeficiency virus in Bangkok, Thailand. *Annals of the Academy of Medicine, Singapore*.

[32] Thompson D. S., Bain B., East-Innis A. (2008). The prevalence of mucocutaneous disorders among HIV-positive patients attending an out-patient clinic in Kingston, Jamaica. *West Indian Medical Journal*.

[33] Wiwanitkit V. (2004). Prevalence of dermatological disorders in Thai HIV-infected patients correlated with different CD4 lymphocyte count statuses: a note on 120 cases. *International Journal of Dermatology*.

[34] Goldstein B., Berman B., Sukenik E., Frankel S. J. (1997). Correlation of skin disorders with CD4 lymphocyte counts in patients with HIV/AIDS. *Journal of the American Academy of Dermatology*.

[35] Boonchai W., Laohasrisakul R., Manonukul J., Kulthanan K. (1999). Pruritic papular eruption in HIV seropositive patients: a cutaneous marker for immunosuppression. *International Journal of Dermatology*.

[36] Obuekwe O. N., Onunu A. N. (2006). Gender and oral manifestations of HIV infection among adult Nigerians. *African Journal of Reproductive Health*.

[37] Onunu A. N., Obuekwe N. (2002). HIV-related oral diseases in Benin City, Nigeria.. *West African Journal of Medicine*.

[38] Shittu R. O., Adeyemi M. F., Odeigah L. O., Mahmoud A. O., Biliaminu S. A., Nyamngee A. A. (2013). Prevalence and pattern of cutaneous lesions in relationship to CD4 cell counts among newly diagnosed HIV patients in University of Ilorin Teaching Hospital (UITH), Ilorin, Nigeria. *Oral Health and Dental Management*.

[39] Ukpebor M., Braimoh O. B. (2007). HIV/AIDS; oral complications and challenges, the Nigerian experience. *Benin Journal of Postgraduate Medicine*.

[40] Sharma Y. K., Sawhney M. P. S., Bhakuni D. S., Gera V. (2004). Orocutaneous manifestations as markers of disease progression in HIV infection in Indian setting. *Medical Journal Armed Forces India*.

[41] Puttaiah K. S., Sunith V., Mallikarjun A. (2010). A hospital based cross sectional study of mucocutaneous manifestations in the HIV infected. *International Journal of Collaborative Research on Internal Medicine and Public Health*.

